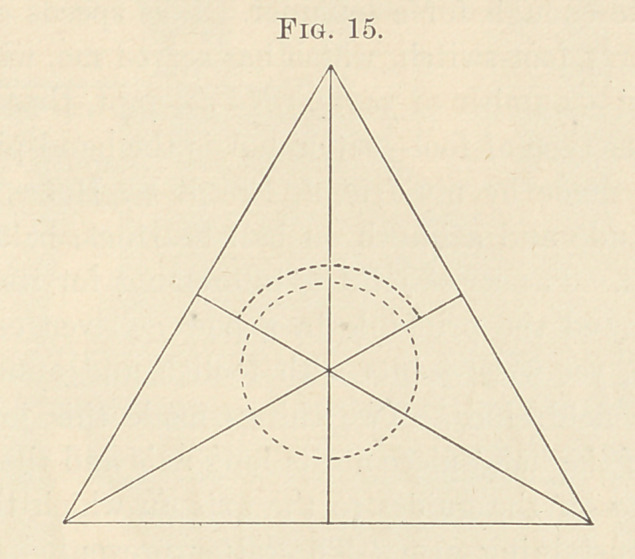# Common-Sense Occlusion, or “Bite”

**Published:** 1900-04

**Authors:** W. Warrington Evans

**Affiliations:** Washington, D. C.


### International Dental Journal.

VOL. XXI. APRIL, 1900. NO. 4.

### Original Communications.[1]

### COMMON-SENSE OCCLUSION, OR "BITE."[2]

BY W. WARRINGTON EVANS, M.D., D.D.S., WASHINGTON, D. C.

THIS subject is one that has been discussed in journals, text-books, and conventions, and yet the majority of dentists are as ignorant of the matter to-day as they were thirty years ago.

While I may not be able to present my method as comprehensively as many would desire,—and it is difficult to do without practical demonstration,—yet I will state that it is not complicated by either mechanism or patents on articulators, and I have nothing to sell. My object is, if possible, to assist my fellows in what is generally considered a difficult matter,—*i.e.*, the construction of artificial dentures.

My results have been highly satisfactory for the last thirty-odd years, during which time I have frequently taken impressions and occlusions within the space of an hour or two, for patients leaving or passing through the city, and sent the finished work to them, feeling assured that the dentures would require no grinding or adjustment, the most conclusive proofs of which have been complimentary letters, enclosing checks.

[1] The editor and publishers are not responsible for the views of authors of papers published in this department, nor for any claim to novelty, or otherwise, that may be made by them. No papers will be received for this department that have appeared in any other journal published in the country.

[2] Read before the Academy of Stomatology, December 26, 1899.

I likewise desire to state, in the beginning, that it is not my intention to criticise any other methods that have been presented, or to be especially accurate in my nomenclature, but will reduce the subject to simple language and make it as practical as possible.

A correct *occlusion,* or " bite," as I understand it, is the obtaining the correct distance between the alveoli and the relative position the jaws had occupied, closed normally, before the loss of the natural organs, as shown in Fig. 1.

Fig. 1.

To better understand the importance of accuracy and the difficulties in our way, it may be well to consider in a few words the anatomical relations.

The superior maxilla being fixed, it is not necessary to consider it, but the inferior maxilla, or " mandible," being movable and controlled by both lateral and vertical ligaments and muscles influenced by temperament, causes much trouble and annoyance, and must be met by positive system.

The condyles of the mandible articulate with the anterior portion of the glenoid fossa of the temporal bone, forming the temporomaxillary articulation. When the condyles are in normal articulation with the glenoid fossa, then we have a normal occlusion. As in all joints of the human system, here we find the interarticular cartilage covering the fossa and the condyles, to give free motion.

The movements of the mandible are governed by three ligaments and four muscles, as follows: The capsular, spheno-maxillary, and the stylo-maxillary ligaments, and the temporal, masseter, internal and external pterygoid muscles. The three first-named muscles, with the ligaments, control the vertical motion, and the external pterygoid gives the lateral or grinding motion to the jaw.

These ligaments and muscles are under no unusual strain when the natural organs are present in the oral cavity and normally occluded. But as soon as the teeth are lost there is naturally an unusual amount of strain on these ligaments and muscles, to maintain the normal temporo-maxillary articulation, thus creating what is known as "jimber jaw" and unnatural protrusion of the mandible. It is with this knowledge before us, and a comprehension of temperamental diagnosis (which also influences the muscular action of the mandible), that we see the necessity for taking time and care to obtain a correct occlusion of the teeth.

Having described the anatomical relations of the mandible, it would be unjust to pass unnoticed the valuable "natural law of the equilateral triangle" of the mandible, discovered and recorded by our late hard-working brother, Dr. W. G. A. Bonwill. His greatest mistake rested in the fact that he believed this law could only be made useful by his articulator, thus preventing a just appreciation of a most valuable discovery.

It is only within the past few months that, having taken up this particular subject to endeavor to find the positive, practical merits therein contained, and to see also if it were worthy of consideration for my book, I found through this study much more of value and practical use which could be derived from it than it had credit for heretofore.

The drawings submitted have been prepared with proportional accuracy and with as few lines as possible for intelligent description.

Dr. Bonwill's claim is, "that from the centre of one condyloid process to the other, four inches is the average; and it will be found that from this same centre of the condyloid process to the median line at the point where the inferior centrals touch at the cutting edge, is also four inches." My own investigations at the government museums as to the measurements, etc., substantially sustain his arguments.

As my object in presenting these drawings of the equilateral triangle is only for the purpose of establishing the normal form of the human maxillæ, I will refer any one who desires a fuller description of Dr. Bonwill's discovery to his last article on the subject, published in the *Items of Interest* for September, 1899.

FIG. 2.

In Fig. 2 we have a representation of the mandible. *A, A,* the supposed centre of the condyloid process, which, when of normal size, we consider at four inches apart; two lines drawn from *A, A* to *B,* of equal length, make the equilateral triangle; from *B* to a point midway between *A, A* gives a middle or vertical line, *C.* By measuring the united diameters of the three inferior front teeth, from the mesial surface of the central incisor to the distal surface of the canine, same side, at the widest point, with a pair of dividers, and placing one point of the dividers at *B*, the other on the vertical line, at *D*, describe a circle, *D* being the central point. From *A, A* describe two arcs of a circle, from *D* to *E, E;* these, with the vertical line, will divide the circle into three equal parts. That portion running from *E, E* through *B* will be the exact space to be occupied by the six front teeth.

A line drawn from *e* to *e*, and extended down to a point on the vertical line to form a small equilateral triangle, will locate *F*, which will give the size of the first bicuspid, if an arc is described, pivoting at *A, A*, from *F* to *G, G*.

Fig. 3.

To obtain the size of the second bicuspid, which is larger, place one end of the dividers at *A, A*, and describe an arc from *H* (the intersection of the circle with the vertical line) to *I, I*.

The space for the first molar is obtained by measuring the distance from *F* to the point on vertical line where it is crossed by the line from *e* to *e,* and measuring this same distance on vertical line from *H* to *J;* then describe your arc from *J* to *K,* pivoting at *A.*

The distal surface of the second molar is obtained by measuring the same distance on vertical line as from *H* to *J,* establishing *L.* From *L* another arc is described to *M,* pivoting always from *A* or the condyle.

A line should now be drawn from *A* to the anterior distal surface of the canine, which should pass through the buccal cusps of the bicuspids and molars.

The *superior maxilla* differs somewhat in its lines, owing to the larger circle required for the six front teeth and size of the first bicuspid (Fig. 3).

Experimenting for some time, I found that after describing a larger circle, as laid down by Dr. Bonwill, the only reliable guide to obtain the exact size of the first bicuspid was to measure the diameter of the lateral incisor.

This, I discovered, if in a normal condition, always had exactly the same diameter as the first bicuspid. I therefore measured the diameter of the lateral incisor with the dividers, and, placing one point at *E* (Fig. 5), or distal surface of the canine, measured down the line from *E* to *A,* and described an arc from *F* to *G,* pivoting at *A.*

To get the distal surface of the second bicuspid, take the distance from *D* to *G,* and extend down the vertical line to *H,* and describe an arc from *H* to *I,* from the condyle.

The molars are obtained by measuring with dividers from *D* to *H,* and using the same measurements on the vertical line from *H* to *J,* and from *J* to *L,* describing arcs from *J* and *L* to *K* and *M,* pivoting at *A.*

In the superior maxilla, the line from *E* to *A,* instead of running through the buccal cusps of the bicuspids and molars, should barely touch their buccal faces.

While a knowledge of the equilateral triangle is a desirable feature, and of assistance to the dental artist in the arrangement of artificial teeth as substitutes for the natural organs, and to the manufacturer for proper proportioning, it is secondary to the manner of taking a correct occlusion, which it is my main object in this paper to make clear.

It matters little whether the human jaw be established on lines of an equilateral triangle or a trapezoidal square, if the student or dentist, desiring a perfect occlusion, can derive no practical benefit from these scientific principles, but can rely implicitly, if he have the proper knowledge of manipulation, upon a simple piece of gutta-percha.

To obtain a perfect occlusion, there are *three things* to be considered:

1. A material to make the articulating base-plate with, that will be plastic to work and sufficiently rigid to hold its form when moulded and carved to suit the case, and which will withstand the heat of the mouth without undergoing change.

2. The plate or plates should be so shaped and smoothed as to be light, and to conform as nearly as possible to the finished denture, allowing for length of teeth, but without unnecessary bulk.

3. When satisfied that the bite is correct, find a means to lock the plates, if in an edentulous mouth, so that when removed the perfect occlusion may be retained without disturbance.

If these rules are strictly adhered to, as hereafter described in detail, there will be no need for mechanical articulators, nor will it be necessary to see the patient again until the work is completed, when it may be placed in the mouth without touching with the file or grindstone.

After carefully testing *all* the materials, to find *which* will meet the above requirements, the writer prefers the use of gutta-percha, as having the greatest number of advantages. The difficulties, however, that have been advanced are, that it requires too high a heat to soften it, is too hot to handle, and that it shrinks too much. This is the trouble when first purchased from the manufacturer, but if one will boil it a few minutes, working a little "gutta-percha and wax" compound with it, hammer it out on the anvil, and boil two or three times more, he will make it plastic, comparatively non-shrinkable, and softening at a lower temperature than modelling compound. It will mould like putty. Work up a half-pound until it becomes as described, and by saving the cuttings and adding a new sheet every two or three weeks, to keep up stock and quality, you will always have a sufficient amount of the proper material on hand.

For an upper denture, soften a cake of gutta-percha in hot water, form it into a ball about the size of a small walnut, and lay upon the centre of the model; after having soaked the model to saturation in cold water, work the ball with the thumbs (Fig. 4)

FIG. 4.

FIG. 5.

FIG. 6.

FIG. 7.

over the surface of the model, first to a thin edge extending back nearly to the soft palate, next forward and to the sides over the alveolar ridge. Taper as high up over the ridge as you expect to carry the plate labially and under the buccinator muscles, finally working all the surplus perpendicular with the ridge and outer margins of plate front and sides (Fig. 5). Thin the plate well out from the centre. In a word, work this plate to conform in shape as nearly as possible to that expected in the finished plate, and make it thin, especially at the heel. Harden by dipping in cold water, and trim ridge, sides, and edge with penknife, as shown in Figs. 6 and 7. This will give you a base-plate that will fit the mouth, feel natural, and one that will enable you to engage your patient in conversation, thus giving you an opportunity to study the facial expression, which is very important in obtaining a correct occlusion. Tell an anecdote, to make the patient smile, as this is the severest test to an artificial denture. These gutta-percha plates should be so trimmed as to virtually represent the finished denture.

Having trimmed off the biting edge of the ridge to a line of uniform contact all around, and having satisfied yourself as to the proper distance of the jaws from each other, contour of plates, etc., mark the median line on the base-plate, chat with the patient, and examine several times to make sure the marked line comes true each

Fig. 8.

time; then cut some V-shaped notches, as shown in Fig. 8, on the cutting edge, soften a small piece of "dainty" wax in summer, and "gutta-percha and wax" in winter, and when quite soft roll to a rope and lay upon the ridge, pressing sufficiently to fix its position. Finally, place in the mouth and have patient bite down through the wax to the base-plate, being careful to observe if the mark of the median line is exactly in the same place as before applying the wax (Fig. 9). Gently work the wax to the fronts of

Fig. 9.

the teeth and against the outside of the base-plate while the patient keeps the jaws closed. Should you fail to have the patient close the jaws exactly at the median line, as before the piece of wax was applied, try again until you succeed. You will be well repaid. Never tell your patient to bite naturally; judge that for yourself.

Fig. 10.

If a full set, make the lower plate in the same way, only work into the soft gutta-percha, while ridging up, a piece of German silver wire of No. 16 wire gauge, to stiffen it. The writer always keeps several pieces of wire, of different sizes and of horseshoe shape, in his box of gutta-percha for both upper and lower cases. This stiffening with wire enables one to trim the base-plate to a nicety, and at the same time prevents the heat of the mouth from softening it out of shape, particularly in summer.

Having procured the occlusion (Fig. 10), wet the back extension of the model and coat the surface with a solution of ethereal soap, as this makes the simplest and most effective medium for separating articulating models. Replace the gutta-percha upon the model carefully (Fig. 11), wet some soft paper, and pack gently

Fig. 11.

into the hollow orifice between the model portion of the plate and the top lines of the bite, the portion that would be occupied by the tongue if in the mouth. This is to prevent the plaster from running into this space and giving trouble. Next, pour the articulating model. Fig. 12 represents a full set occluded by gutta-percha plates before separating; Fig. 13, the articulating models after the plates have been taken off.

There are those, probably, who may consider the backward extension or plaster articulator an obsolete method. It is old, I admit, but not obsolete, so long as it continues to serve the purpose as well as, if not better than, more modern, complicated, and expensive appliances.

FIG. 12.

FIG. 13.

The chief claims are: it is positive in one set position, and there are no screws or bolts to get out of order or be tampered with by an inquisitive friend in a visit to the laboratory.

Your occlusion or bite, as described, if correct, should be so articulated as never to be changed until the work is finished. If it is not correct, no mechanical articulators now constructed will ever make it so perfect as not to require after-grinding or fitting.

A few words more, and I am through; but before closing I desire to present two more illustrations bearing upon the same subject. One is a sectional view (Fig. 14) of what I call normal

Fig. 14.

occlusion, or the line of force considered by degrees, running from a meridian at cutting edge, through crown and root to apex of root. I figure it thus: Upper central incisor, eighty degrees; lower central incisor, eighty-five degrees; upper lateral incisor, eighty degrees; lower lateral incisor, eighty-five degrees; upper canine, eighty-five degrees; lower canine, ninety degrees; the same for the upper and lower first and second bicuspids, while the line of force of the molars is a direct eighty-five degrees running straight through. If in error, I desire to be corrected, as this is only a portion of much more valuable matter.

Finally, in Fig. 15 we have the possible key to the utility of Dr. Bonwill's equilateral triangle. By keeping in our operating-room little tablets, printed in this form, without the dotted circles, we may have a useful means of getting at the correct size or width of teeth of any mouth we desire to furnish with an artificial denture. If there are teeth in the lower jaw and none in the upper, measure the three lower anterior teeth on one side, allowing for any irregularity, from mesial surface of the central to the distal surface of

Fig. 15.

the canine, with dividers; and describe a circle on tablet diagram. Extend the dividers one-eighth of an inch, and describe the third of a circle, as shown on diagram, and you may rely upon it that any six front teeth which will just fit around this arc of a circle are of the proper size and will suit that particular case. Likewise, in an entirely edentulous mouth, measure with the dividers, seeking a centre that will form a circle touching just outside of the alveolar ridge, in front and on side, at the supposed point just back of the canines, and describe circles on diagram, and you have like result. This a servant can take to a dental depot, or it can be mailed by a country practitioner. This is the practical side of the triangle, to say nothing of its usefulness in setting up teeth, etc., which we have not time now to discuss.

## Figures and Tables

**Fig. 1. f1:**
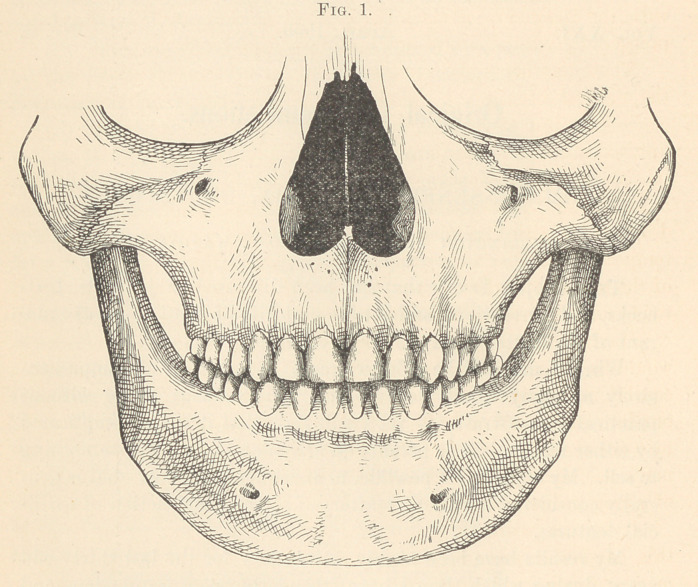


**Fig. 2. f2:**
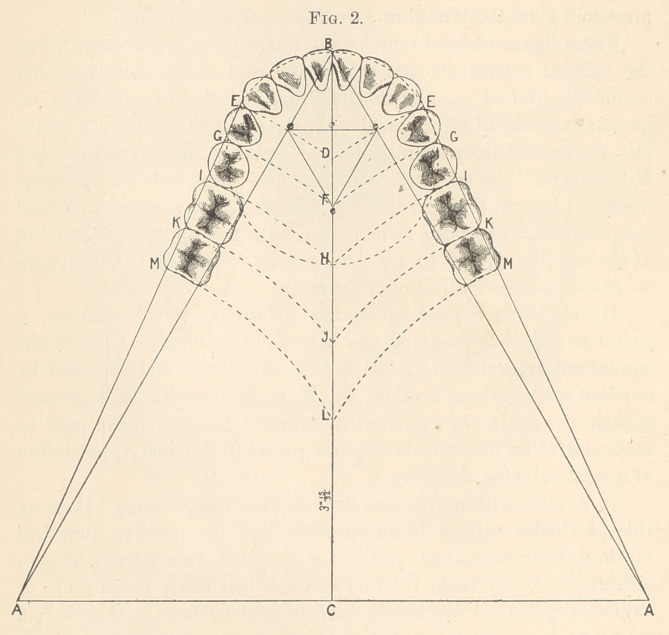


**Fig. 3. f3:**
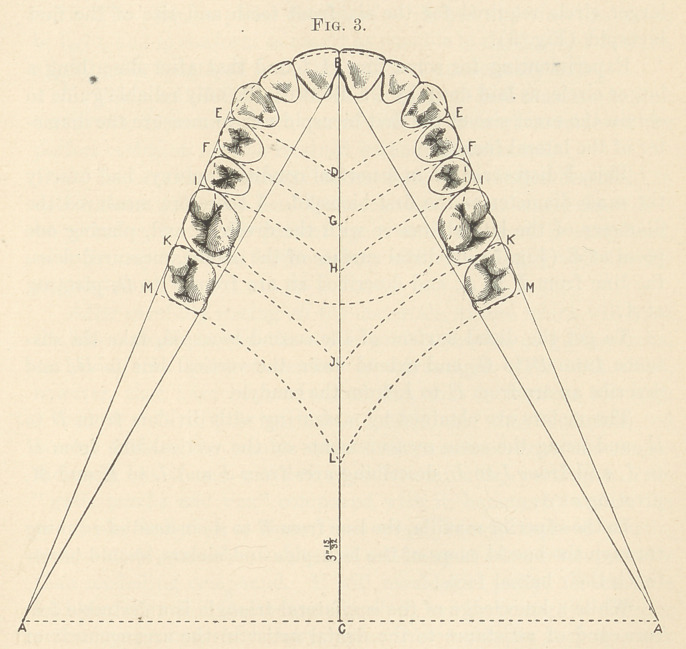


**Fig. 4. f4:**
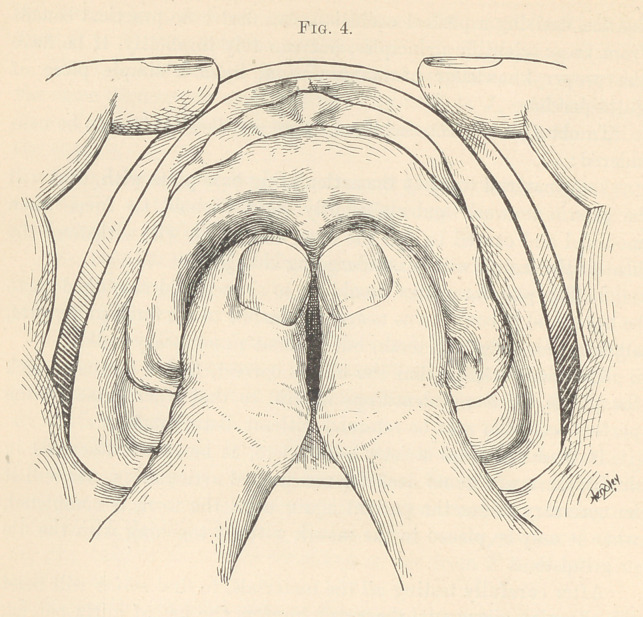


**Fig. 5. f5:**
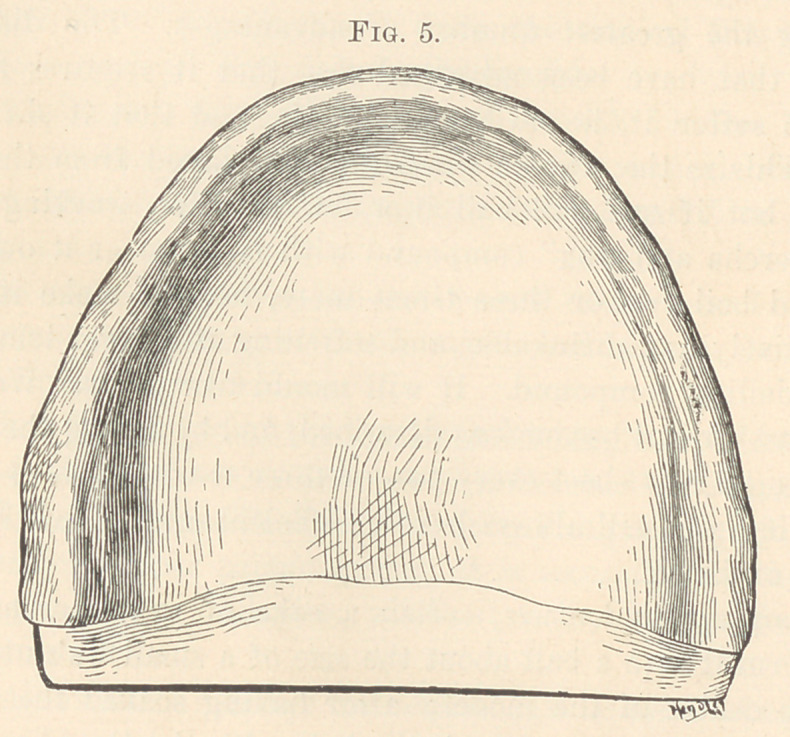


**Fig. 6. f6:**
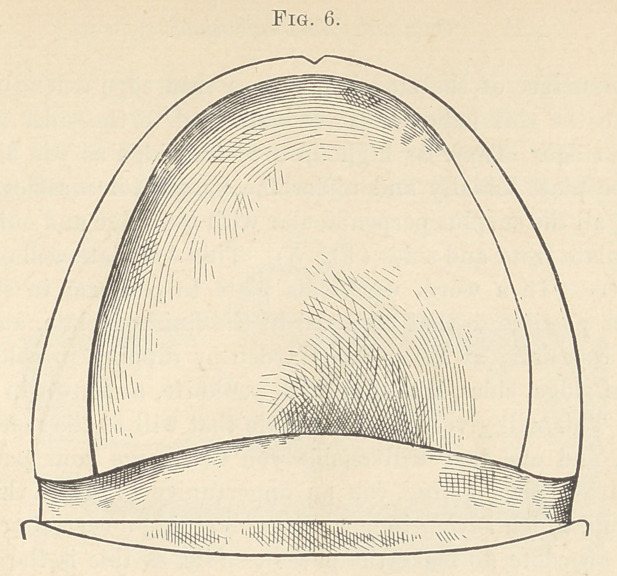


**Fig. 7. f7:**
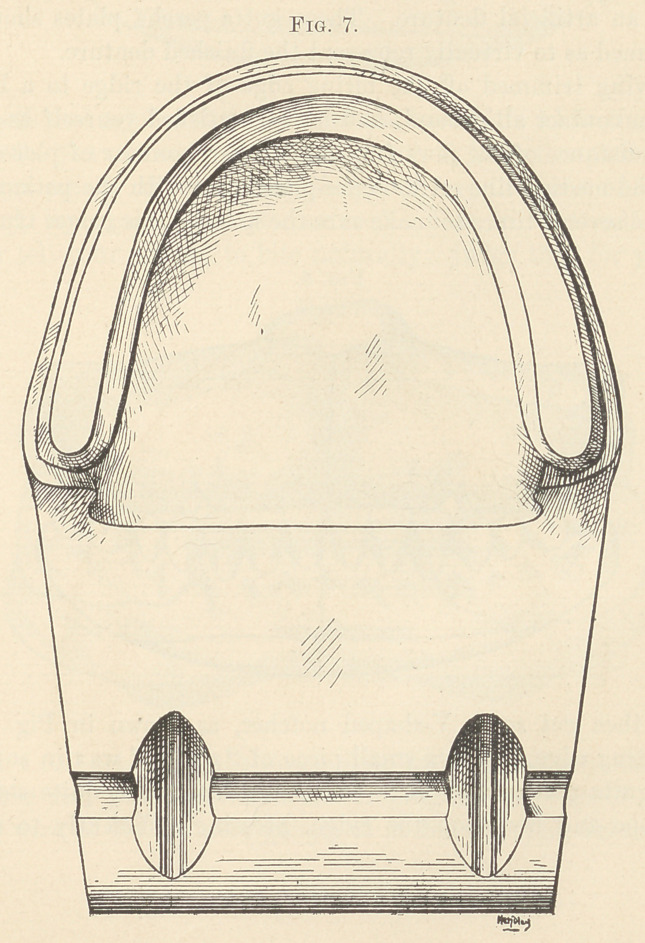


**Fig. 8. f8:**
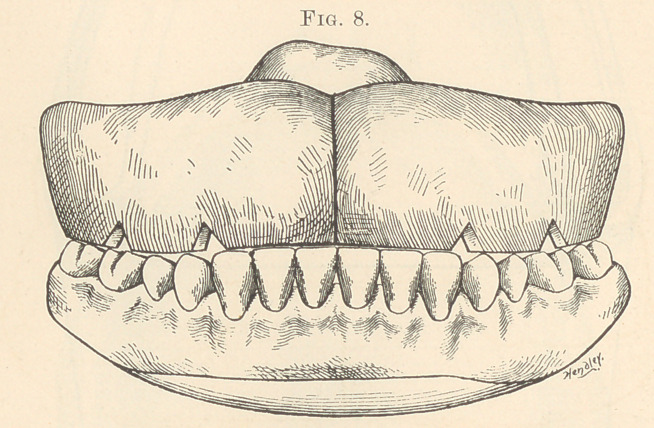


**Fig. 9. f9:**
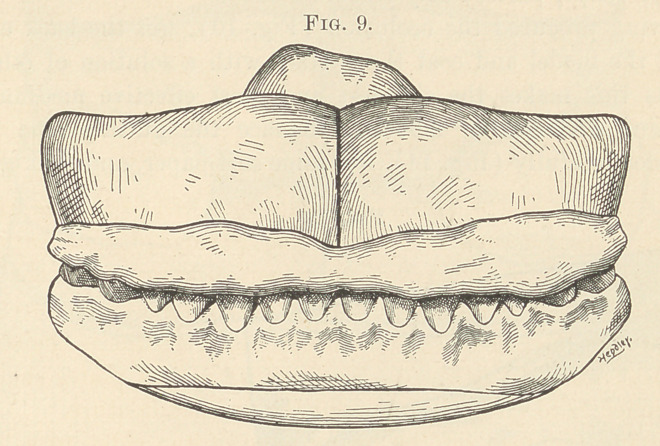


**Fig. 10. f10:**
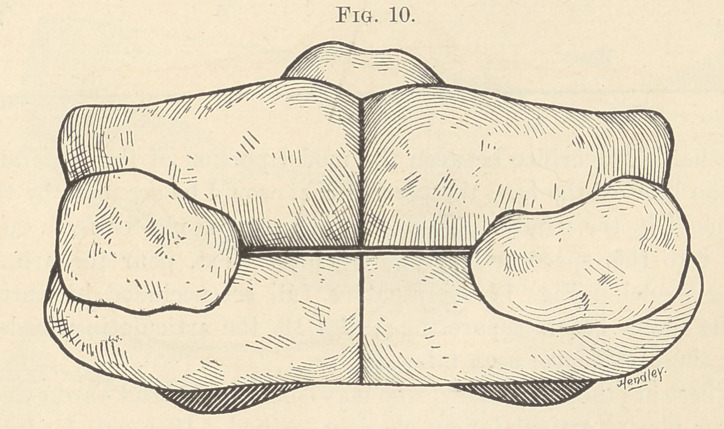


**Fig. 11. f11:**
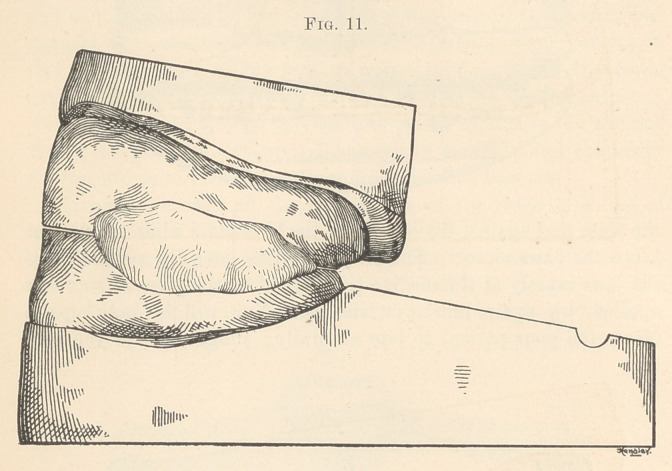


**Fig. 12. f12:**
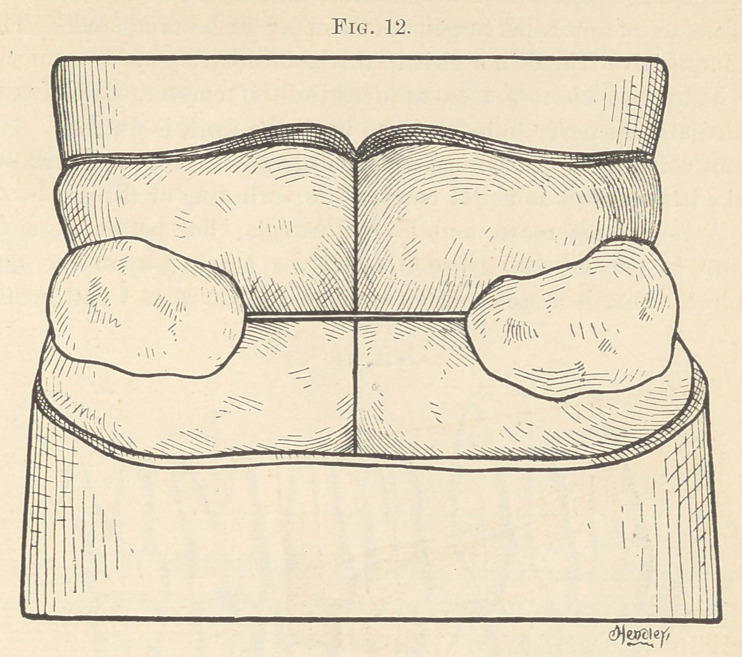


**Fig. 13. f13:**
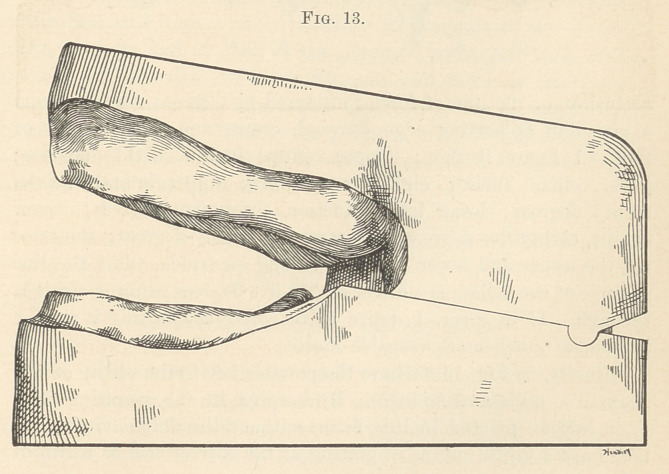


**Fig. 14. f14:**
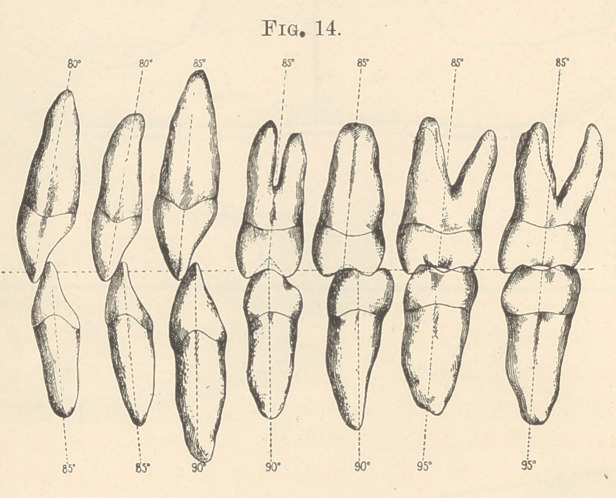


**Fig. 15. f15:**